# Prevalence of key potentially inappropriate drugs use in pediatrics: a cross-sectional study

**DOI:** 10.1186/s12887-024-04921-w

**Published:** 2024-07-09

**Authors:** Mariam Jihad Diab, Sham ZainAlAbdin, Salah Aburuz, Rami Beiram, Amal Akour, Anan Jarab, Tasnim Dawoud

**Affiliations:** 1 Department of Pharmacology and Therapeutics, College of Medicine and Health Sciences, United Arab Emirates University, Al-Ain, United Arab Emirates; 2 Department of Biopharmaceutics and Clinical Pharmacy, School of Pharmacy, The University of Jordan, Amman, Jordan; 3 College of Pharmacy, Al Ain University, Abu Dhabi, United Arab Emirates; 4 Pharmacy Department, Sheikh Khalifa Medical City, Abu-Dhabi, United Arab Emirates; 5 Department of Clinical Pharmacy, Faculty of Pharmacy, Jordan University of Science and Technology, Irbid, Jordan

**Keywords:** KIDs list, Pediatric, Inappropriate medication use, Pediatric Pharmacy Association

## Abstract

**Background:**

Appropriate drug prescribing in the pediatric population is challenging, given this age group’s unique pharmacokinetics. This has inevitably led to a high incidence of adverse drug reactions in this population. To address this issue, the Pediatric Pharmacy Association (PPA) compiled a list of 67 drugs that are inappropriately used in the pediatric population called the Key Potentially Inappropriate Drugs “KIDs list”.

**Aim:**

To estimate the prevalence of potentially inappropriate medications (PIM) use in pediatric wards based on the KIDs list criteria.

**Methods:**

A retrospective observational study was conducted and included pediatric patients who were admitted to five pediatric wards during 3 years (2019–2021). The drugs in the KIDs list were matched to the hospital formulary and 11 matched drugs were included in the study. For each individual drug, the patient’s electronic file was reviewed to determine the prescription appropriateness according to the KIDs list criteria.

**Results:**

Among 3,166 pediatric patients admitted to pediatric wards, a total of 415 patients received a PIM listed in the KIDs list. The mean age was 8.6 (± 4.9) years old, and 60.0% (*n* = 251/415) were males. The overall prevalence of PIM use was 13.0% (*n* = 415/3166). Among the prescribed PIM, metoclopramide was the most commonly prescribed PIM 42.8% (*n* = 178/415), followed by tramadol 37.6% (*n* = 156/415).

**Conclusion:**

Given the high prevalence of inappropriate medication use in the pediatric wards, future research on strategies and interventions should be recommended to limit the use of PIMs and provide safer alternatives for the pediatric population.

## Introduction

Appropriate drug prescribing in the pediatric population is challenging due to the distinctive pharmacokinetics that this age group possesses [1]. Historically, children have been under-represented in clinical trials [2–4], making it hard for clinicians to make evidence-based decisions regarding their care. The World Health Organization (WHO) reported that half of all medications are inappropriately prescribed [5]. Unfortunately, the extent of inappropriate prescribing has not been well studied among pediatric population [6], which presents a challenge to clinicians due to lack of a clear evidence-based guidance on the appropriate utilization of medications in children.

The lack of this information has driven physicians to prescribe medications in an off-label manner, with no strong evidence of safe use. This has inevitably led to a high incidence of adverse drug reactions (ADR) in this vulnerable population, as reported by several studies in the past decade [7–11]. A previous prospective observational study reported that ADRs were responsible of 4.0% of the hospital admissions to a British children’s hospital, and 33.0% of these ADRs were possibly avoidable [10].

The Beers Criteria for Potentially Inappropriate Medication Use in Older Adults, first published in 1991 [12], has been used for over two decades by the health care providers to identify Potentially Inappropriate Medication (PIM) use in elderly patients. Multiple studies have used the Beer’s list to identify the prevalence of PIM use in the elderly [13–16]. Accordingly, this tool has helped health care providers in identifying the gaps in practice and implement appropriate interventions to mitigate the use of PIM in the elderly population [17–19]. Given the Beer’s criteria’s great impact on the proper and safe use of medications among elderly, a similar list for the pediatric population was greatly needed.

In 2020, the Pediatric Pharmacist Association (PPA), an international organization that has successfully collaborated with ACCP, APhA, and ASHP to create an initiative that officially establishes pediatric pharmacy as a specialized field [20], compiled a list of drugs that should be avoided or used in caution among pediatrics. The Key Potentially Inappropriate Drugs in Pediatrics, commonly known as the “KIDs List”, includes 67 drugs and 10 excipients. The PPA has introduced this list to be used a tool to evaluate and enhance the quality of care, decrease costs, and identify gaps in research and practice the pediatric population. Interestingly, the KIDs list includes the rationale for inclusion as well as strength of recommendation and quality of evidence for each drug to be used among pediatrics. Given the lack of high-quality literature addressing the use of medications in the pediatric population, many of the recommendations in the KIDs list are considered weak. Hence, the KIDs list doesn’t provide contraindications, and clinicians are advised to consider the whole clinical picture when making decisions regarding the use of these drugs [21].

To best of our knowledge, no study has yet investigated the prevalence of potentially inappropriate medication (PIM) use among pediatrics using the KIDs list in the Middle East. There is an urgent need for targeted interventions and improved awareness among healthcare professionals to enhance medication safety and prescribing practices in the pediatric population.

## Method

### Aim

The aim of this study is to identify and estimate the prevalence of PIM use among pediatric patients admitted to the pediatric and neonatal wards at Tawam hospital, United Arab Emirates based on the KIDs list criteria.

### Study design and setting

This is a retrospective cross-sectional observational study of the potentially inappropriate medication use in the pediatric wards during the duration ranging from January 2019 to December 2021. Tawam hospital is a tertiary 509-bed hospital that offers both adult and pediatric care. It offers specialized pediatric and neonatal care services and has 129 pediatric beds (36 medical pediatric beds, 18 pediatric oncology, 19 pediatric surgery, and 41 neonatal intensive care beds). Tawam hospital is the largest hospital offering pediatric services in Al Ain (with a population of over 750,000 people), hence it experiences a significant patient load [22].

### Subject selection and sampling

#### Inclusion and exclusion criteria

This study included patients aged ≤ 16 years old, who were admitted to any pediatric ward; pediatric intensive care unit (PICU), pediatric medical (PMED), pediatric surgical (PSURG), pediatric oncology (PONC) and neonatal unit (NNU) at Tawam hospital during January 2019 and December2021. Only patients who received any drug listed in the KID’s list and was inappropriately used (prescribed and administered) according to the KIDs list were included.

On the other hand, the study excluded the consideration of excipients mentioned in the KID’s List, since there is no comprehensive list of medications containing excipients such benzyl alcohol, ethanol, propylene glycol, and others at our hospital.

### Ethical considerations

The study protocol was approved by the Research Ethic Committee at Tawam hospital (Reference number: MF2058-2021-811, 16th November 2021). Given the nature of this retrospective observational study, the study was exempted from the informed consent form. Data was collected without any personal information of the patients and they were identified by the MRN in the hospital system by the primary researcher, who was able to access the medical records.

### Study procedures

Patients’ data was extracted from the medical report and medications mentioned in the KIDs list were matched with hospital formulary. After that, a report was generated for each medication after reviewing the patient’s medical record. Each report represents the appropriateness of the prescribed medication whether it was appropriately prescribed or not based on the recommendations provided in the KIDs list.

### Study variables and outcomes measures

Collected data includes patient’s demographics (age and gender as well as clinical information (prescribed medication, date of prescribing, and ward). The primary outcome was the prevalence of PIM use at a Tawam hospital during the years 2019–2021 according to the recommendation in the KIDs list. The prevalence of inappropriately prescribed medications was determined by calculating the total number of PIM use among the total reviewed medical records that included medications mentioned in the KIDs list during the years 2019–2021. Adverse drug reactions (ADRs) were identified using the ADR reporting system at the hospital to determine the prevalence of ADRs from the selected prescriptions. The ADR reporting system at Tawam hospital is a voluntary digital reporting system that uses manual assessment through the Naranjo score. The secondary outcome was the incidence of ADRs reported from the selected prescriptions accordingly.

### Statistical analysis

Data analysis was performed using the Statistical Package of Social Sciences (SPSS) Software (Version 26.0). Categorical data including demographics (gender and age category), prevalence of inappropriately prescribed medications in each pediatric ward and overall, as well as number of cases for each medication that was inappropriately use were described as frequency (percentage), while continues data age was presented as mean (± standard deviation).

## Results

In this study, total of 3166 pediatric patients (age ≤ 16 years old) admitted to any pediatric ward during a period ranging from 2019 to 2021 were identified. Out of them, 415 patients were found to be prescribed inappropriate medications according to the KIDs List. The mean age of the selected patients was 8.6 ± 4.9 years old and majority of them were males (60.0%, *n* = 251). Table 1 shows the demographic characteristics of the studied patients.

**Table 1:** Demographic characteristics of patients receiving PIMs (*n* = 415)

Variables	Mean ± SD
Age, mean (± SD)	8.6 ± 4.9
	***N (%)***
**Gender**	
Male	251 (60.0)
Female	164 (39.5)
**Age Distribution According to KIDs List Classification**	
Neonates < 1 month	21 (5.0)
Neonates < 1 month (Very Low Birth Weight < 1500 g)	19 (4.6)
Infants 1–24 months	41 (9.8)
Children 2–16 years *Pre-school age (> 1 year-5 years)* *School age (> 5 years-12 years)* *Adolescent (> 12)*	334 (80.5) 72 (17.3) 156 (37.6) 153 (36.9)

Table 2 illustrates the prevalence of PIM use. Out of 67 medications mentioned in the KIDs list, 44 medications were matched with the hospital formulary (our formulary is a comprehensive medication list that we use to prescribe medications. There are no patients who receive any other non-formulary medications in our institution) and upon reviewing the 3166 patients’ medical records since 2019 to 2021, 20 medications were identified. Out of the 20 medications identified, only 11 medications were found to be inappropriately used in 415 patients. Therefore, the overall prevalence of use of PIM was 13.0% (*n =* 415/3166). Among the prescribed drugs that have been matched with the KIDs list, metoclopramide (98.0%, *n* = 181/181), Tramadol (100.0%, *n* = 156/156), and Lamotrigine (100.0%, *n* = 25/25) were the most commonly prescribed medications inappropriately. Further details about the prevalence of PIM, recommendations, rationale of the recommendation/ Risk, strength of the recommendation, and quality of the evidence are presented in Table 2.

**Table 2:** Prevalence of Inappropriate Drug Use (*n* = 3166)

Drug	*Total use of each medication (out of * *n* * = 3166)*	Total inappropriate use (*n*)	Prevalence of PIM out of total medication used (%)	*Prevalence of PIM (%) out of * *n = 3166*	Recommendation	Rationale of the recommendation/ Risks	Strength of recommendation	Quality of Evidence
Daptomycin	5	1	20.0%	0.03%	Caution in < 1 year	Neuromuscular and skeletal adverse events	Weak	Very low
Metoclopramide	181	181	100%	5.70%	Avoid in infants, caution in children	Acute dystonia (dyskinesia); increased risk of respiratory depression, extravasation, and death with intravenous use	Weak	Moderate
Haloperidol	1	1	100.0%	0.03%	Avoid in infants, caution in children		Strong	Moderate
Promethazine	3	3	100.0%	0.09%				
Lamotrigine	25	25	100.0%	0.79%	Caution in children; titration needed	Serious skin rashes	Strong	High
Azithromycin	333	1	0.3%	0.03%	Avoid in neonates, unless treating Bordetella pertussis or chlamydia trachomatis	Hypertrophic pyloric stenosis	Strong	High
Midazolam	832	19	2.3%	0.60%	Avoid in very low birth weight neonates	Severe intraventricular hemorrhage, periventricular leukomalacia, or death	Strong	High
Olanzapine	3	3	100.0%	0.09%	Caution long-term use (> 24 weeks) in children	Metabolic syndrome (weight gain, hyperlipidemia, hyperglycemia)	Strong	High
Sodium phosphate enema	267	16	6.0%	0.50%	Avoid in infants	Electrolyte abnormalities, acute kidney injury, arrhythmia, death	Strong	High
Tramadol	156	156	100.0%	4.93%	Caution in children unless pharmacogenomic testing is used	Respiratory depression	Weak	Low
Valproic acid	44	12	27.0%	0.38%	Avoid in infants Caution in < 6 years	Pancreatitis, fatal hepatotoxicity	Strong	High

Table 3 represents the prevalence of inappropriate prescriptions compared to the prevalence of appropriate prescriptions in each pediatric ward. The pediatric oncology ward was accounted for the highest number of inappropriate prescriptions, where 36.0% (*n* = 147/410) of prescriptions in the PONC were identified to be inappropriately used. This was followed by the neonatal unit (NNU) which shows 23.1% (*n* = 21/91) of prescriptions being inappropriate.

**Table 3:** Distribution of paediatric patients received inappropriate medications by ward

Ward	Total pediatrics’ records screened *(n = 3166)*	Appropriate use of medications based on KID’s list *(n = 263)*	Inappropriate use of medications based on KID’s list *(n = 415)*	Inappropriate use of medications based on KID’s list (%)
Neonatal Unit (NNU)	91	70	21	23.1
Pediatric Intensive Care Unit(PICU)	827	814	13	1.6
Pediatric Medical (PMED)	1321	1192	129	9.8
Pediatric Surgical (PSURG)	517	412	105	20.3
Pediatric Oncology (PONC)	410	263	147	35.9

**Fig. 1:**
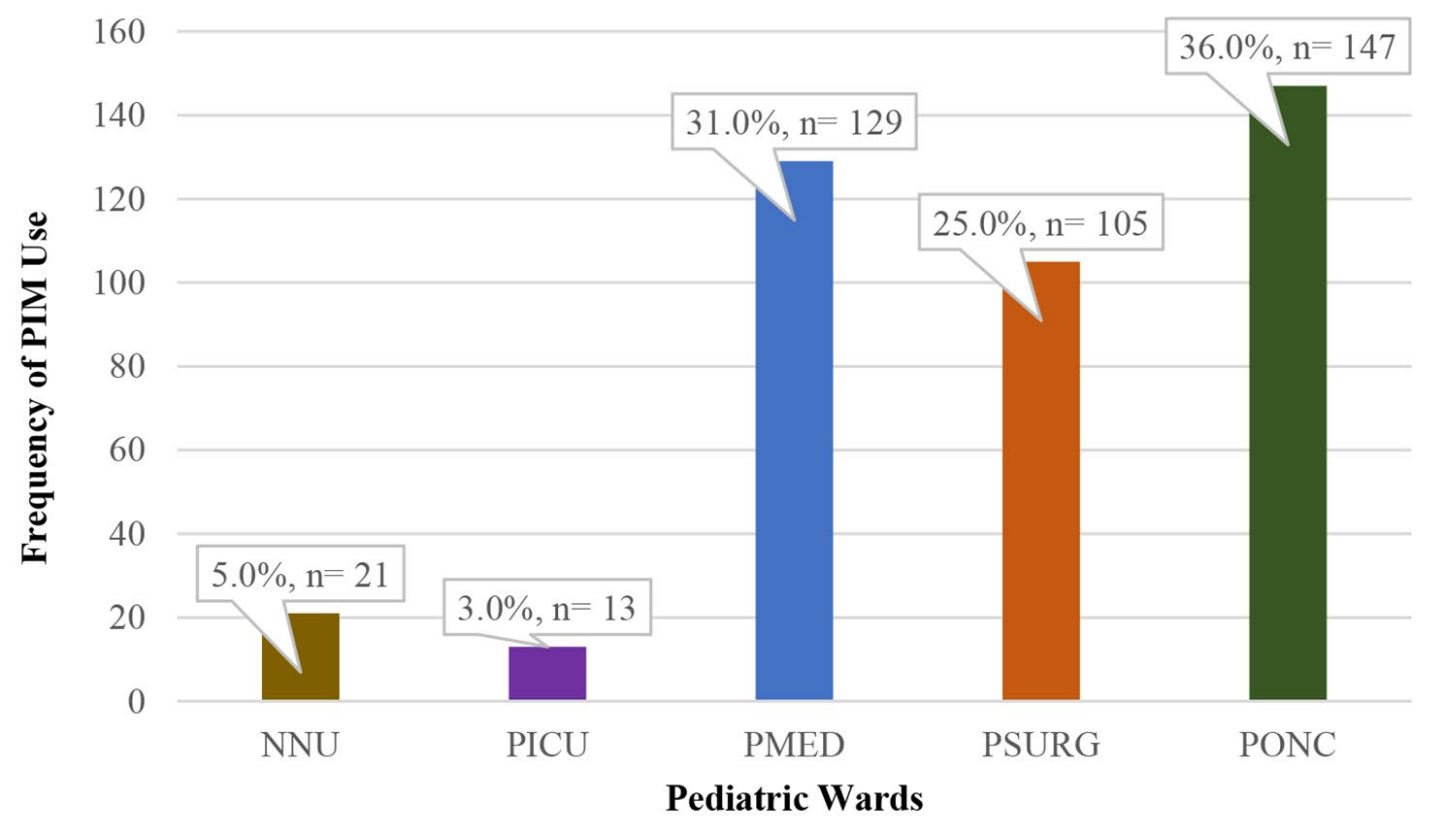
Distribution of Inappropriate Medication Use by Ward (*n* = 415)

**Fig. 2:**
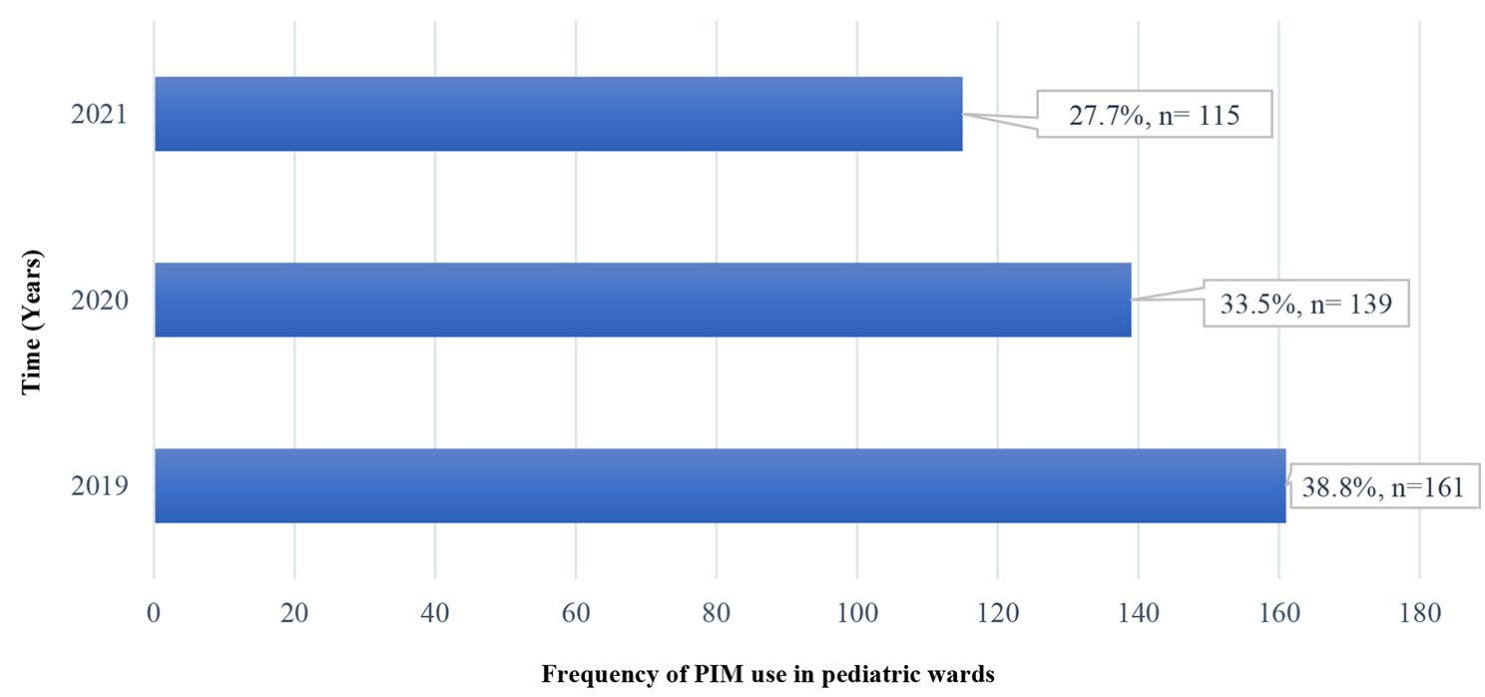
Prevalence of PIM use in pediatric wards by year (*n* = 415)

Figure 1 displays the distribution of cases that had received PIM in the pediatric wards. Pediatric oncology and surgical wards included most of cases who were prescribed PIM ((35.0%, *n* = 149/415), and (31.0%, *n* = 128/415); respectively). Figure 2 represents the prevalence of PIM use in pediatric wards during the studied period. In addition, no adverse drug reactions were reported from any of the inappropriate prescriptions identified during the period from 2019 to 2021.

## Discussion

The present study is the first in the world to estimate the prevalence of potentially inappropriate medication (PIM) use among pediatric patients admitted to the Hospital based on the 2020 KIDs list criteria [21]. The prevalence of PIMs in Tawam hospital between 2019 and 2021 was 13.0%, with the highest prevalence observed among pediatric oncology patients. The most commonly prescribed PIM was metoclopramide, which was used in infants and children for the treatment of nausea and vomiting, increasing the risk of acute dystonia and respiratory depression [23]. The second most commonly prescribed PIM was tramadol, commonly used for post-operative pain management, which carries an increased risk of respiratory depression in children [24]. Both of these medications are commonly used in the oncology ward, since they are indicated for managing adverse drug reactions of the anticancer medications.

In addition, the pediatric units that were commonly prescribing inappropriate medications were pediatric oncology and NNU. These results are expected due to the complexity of the cases encountered in both settings and the lack of available alternatives.

There were no adverse drug reaction reports from the years 2019–2021 for any of the identified inappropriately used medications. It is important to note that adverse drug reactions tend to occur more frequently than documented. Although healthcare professionals are continuously encouraged to report ADRs, a number of challenges exist that prevent this from happening. Some of these challenges include complexity of reporting procedures, lack of time and fear of blame [25].

When interpreting the results of this study, the authors looked into the strength of recommendation and quality of evidence behind the inclusion of each medication in the KIDs list. Out of the 11 drugs identified as PIM in our study, 7 had a strong recommendation against use, given a moderate to high strength of evidence found by researchers. However, the two most common PIMs prescribed at our hospital, metoclopramide and tramadol, had a weak strength of recommendation, and subsequently a low quality of evidence. This explains the decision of the prescribers to use these medications efficiently as there is no strong evidence to prove the risk of using them for pediatric population.

Previous studies used the Beer’s criterion to assess the PIM use in geriatrics. In comparison with our findings, a study conducted at Tawam Hospital in the UAE reported an incidence of PIM use in similar medications to ours; such as metoclopramide, promethazine, and olanzapine [15]. However, it is important to recognize that comparing the KID’s list with the Beer’s criteria poses significant challenges owing to their different target age groups. Moreover, the disparities in the number of recommendations between the two lists stem from variations in the research that substantiates such suggestions.

The first and only other study to date estimating the rate inappropriate prescribing per the KIDs list recommendations was conducted by *Victoria et al.* Unlike our study, their study evaluated the use of safety measures in place for medications on the KIDs. Their results showed low rates of inappropriate prescribing (prevalence was not calculated) of medications in the KIDs list. Similar to our study, no ADRs were identified through their patient safety reports. They also reported low use of safety measures to mitigate the use of these drugs. Their findings will be used as a base in phase 2 of our study, where we aim to implement appropriate safety measures to limit the use of the identified PIM in our hospital [26].

It is important to acknowledge the fact that the KIDs list includes only drugs approved for use in the United States, but some of these drugs listed have been also approved in other countries. Hence, the results should be applied with caution and limitations and formulary alternatives should be considered. For instance, metoclopramide should be avoided in infants and used with caution in children according to KID’s list [21]. Likewise, in 2013, the European Medicine Agency (EMA) recommended against the use of medications containing metoclopramide in the European Union (EU) as part of the global effort to tackle the issue of pediatric medication errors. It is recommended to refrain from using metoclopramide in children under the age of 1 year old. Metoclopramide should only be administered to children over the age of 1 year old as a secondary option at dose not exceeding 0.5 mg/kg/day for a maximum of 5 days. It is used after other therapies have been attempted, for the purpose of preventing delayed nausea and vomiting after chemotherapy, as well as for treating post-operative nausea and vomiting. The EMA has also withdrawn high concentration liquid metoclopramide (> 1 mg/ml) from the market since it has been linked to cases of overdose in children [27].

Additionally, the list may lack other potentially inappropriate medications used in the pediatric population available in different countries as part of their formularies. For example, domperidone, a dopamine antagonist drug is commonly used in our hospital for managing nausea and vomiting as well as treatment of gastroparesis in neonatal and pediatric population, which is not included in the KIDs list and carries similar risk as metoclopramide (QT prolongation) in the same population [28]. However, it would be interesting for the KIDs list to propose alternatives that could be used in children. For example, ondansetron with a favorable safety profile instead of metoclopramide for nausea and vomiting in pediatric oncology or surgical patients, which has minor and self-limiting side effects such as diarrhea [29]. A discussion with all relevant stakeholders including pediatric sub-specialists and pharmacists is necessary to increase their awareness of the KIDs list, and to ensure study results and knowledge are disseminated to better impact patients’ outcomes.

Clinical pharmacists play a significant role in managing inappropriate medication use by identifying and resolving drug therapy problems that can lead to adverse drug reactions [30, 31]. Through services such as medication therapy management, medication reconciliation and chart reviews, clinical pharmacist can identify any potential drug interactions or side effects that may be harmful to the patient. This can prevent medication-related problems, and improve patient outcomes. Collaboration between clinical pharmacists and pediatricians is essential for improving patient care and limiting the use of potentially inappropriate medications [32]. Clinical pharmacists can help in many ways, these include medication therapy management where they review and adjust medication regimens prescribed by pediatricians to optimize drug therapy outcomes. Another way is through medication education by providing information on proper medication use, potential side effects, and drug interactions through workshops and seminars to the medical team. Overall, collaboration between clinical pharmacists and pediatricians is critical for improving patient outcomes and delivering high-quality healthcare in pediatric settings.

Nevertheless, this study is the first study that investigated the prevalence of inappropriate medication use according to the KIDs list criteria. In the next few months, the authors aim to use the results to identify factors associated with PIMs use. Furthermore, collaboration between clinical pharmacists and multidisciplinary pediatric teams is needed to implement appropriate interventions to limit the use of PIMs and to provide guidance on their safe use.

### Strengths and limitations

This study is the first study in the world studying the prevalence of potentially inappropriate medication use using the recommendations in the KIDs list. It included a wide variety of patients with multiple comorbidities, ages, and under different levels of care from general medical up to critical care. This study was done over a period of 3 years, looking at prescribing trends before the KIDs list was published (2019) as well as after (2020–2021). This helped us understand prescribing trends and willingness of the healthcare team to adapt and implement new information available.

This research has some limitations. Firstly, it was a retrospective observational study, relying largely on information documented in the medical records, which are subjected to recall bias and may have some missing clinical information. Secondly, due to time limitations, the authors were not able to study factors associated with PIMs use, which are important in identifying the population who are at greater risk of being prescribed PIMs. Lastly, this study did not evaluate the use of excipients mentioned in the KIDs list. The decision to exclude excipients was made due to lack of complete data on them and the difficulty to identify them in each medication on the formulary. However, we will consider excipients in future research.

## Conclusion

The present study reflects the high prevalence of inappropriate drug prescribing in the pediatric population in our hospital. The study aimed to estimate the prevalence of potentially inappropriate medication use in pediatric wards at a tertiary hospital using the recommendations in the KIDs list. The results showed a prevalence of PIMs of 13.0% between 2019 and 2021, with the highest prevalence observed among pediatric oncology patients. The role of clinical pharmacists in managing inappropriate medication use, identifying and resolving drug therapy problems, and preventing medication-related problems to improve patient outcomes was also highlighted. These findings shall set as a triggering point to commence a comprehensive process to identify and improve appropriate use of medications and safety in the pediatric population.

## Data Availability

Data is Available on request.

## Abbreviations

PPA: Pediatric Pharmacy Association
KIDs: Key Potentially Inappropriate Drugs
PIM: potentially inappropriate medications
WHO: World Health Organization
ADR: adverse drug reactions
PICU: pediatric intensive care unit
PMED: pediatric medical
PSURG: pediatric surgical
PONC: pediatric oncology
NNU: neonatal unit
SPSS: Statistical Package of Social Sciences

## References

[1] Stephenson T (2005). How children’s responses to drugs differ from adults. Br J Clin Pharmacol.

[2] Martinez-Castaldi C, Silverstein M, Bauchner H (2008). Child versus adult research: the gap in high-quality study design. Pediatrics.

[3] Children underrepresented in. drug studies -- ScienceDaily [Internet]. [cited 2021 Nov 9]. https://www.sciencedaily.com/releases/2012/10/121001084119.htm.

[4] Institute of Medicine (US) Committee on Clinical Research Involving Children. Ethical Conduct of Clinical Research Involving Children [Internet]. Field MJ, Behrman RE, editors. Washington (DC): National Academies Press (US). 2004. (The National Academies Collection: Reports funded by National Institutes of Health). http://www.ncbi.nlm.nih.gov/books/NBK25557/. 20669469

[5] Can the Selection and Use of Essential Medicines Decrease Inappropriate Drug. Use? - Reidenberg – 2009 - Clinical Pharmacology & Therapeutics - Wiley Online Library [Internet]. [cited 2021 Nov 9]. https://ascpt.onlinelibrary.wiley.com/doi/abs/10.1038/clpt.2009.10. 10.1038/clpt.2009.10 19451911

[6] Mangione-Smith R, DeCristofaro AH, Setodji CM, Keesey J, Klein DJ, Adams JL (2007). The quality of ambulatory care delivered to children in the United States. N Engl J Med.

[7] Kaguelidou F, Beau-Salinas F, Jonville‐Bera AP, Jacqz‐Aigrain E (2016). Neonatal adverse drug reactions: an analysis of reports to the French pharmacovigilance database. Br J Clin Pharmacol.

[8] Information about adverse. drug reactions reported in children: a qualitative review of empirical studies [Internet]. [cited 2021 Nov 9]. https://www.ncbi.nlm.nih.gov/pmc/articles/PMC2950983/. 10.1111/j.1365-2125.2010.03682.x PMC2950983 20840440

[9] Incidence, characteristics and risk factors of adverse drug reactions in hospitalized children -. a prospective observational cohort study of 6,601 admissions - Citation formats | Research Explorer | The University of Manchester [Internet]. [cited 2021 Nov 9]. https://www.research.manchester.ac.uk/portal/en/publications/incidence-characteristics-and-risk-factors-of-adverse-drug-reactions-in-hospitalized-children--a-prospective-observational-cohort-study-of-6601-admissions(76d4bb75-0456-4476-9e2a-f2d62427cbad)/export.html. 10.1186/1741-7015-11-237 PMC4225679 24228998

[10] Adverse drug reactions causing. admission to a paediatric hospital: a pilot study - Gallagher – 2011 - Journal of Clinical Pharmacy and Therapeutics - Wiley Online Library [Internet]. [cited 2021 Nov 9]. https://onlinelibrary.wiley.com/doi/10.1111/j.1365-2710.2010.01194.x. 10.1111/j.1365-2710.2010.01194.x 21366649

[11] Clavenna A, Bonati M (2009). Adverse drug reactions in childhood: a review of prospective studies and safety alerts. Arch Dis Child.

[12] Beers MH, Ouslander JG, Rollingher I, Reuben DB, Brooks J, Beck JC (1991). Explicit criteria for determining inappropriate medication use in nursing home residents. UCLA Division of Geriatric Medicine. Arch Intern Med.

[13] Chiapella LC, Montemarani Menna J, Marzi M, Mamprin ME (2019). Prevalence of potentially inappropriate medications in older adults in Argentina using Beers criteria and the IFAsPIAM List. Int J Clin Pharm.

[14] Chinthalapudi SS, Cheeti S, Bajpai A, Deepika S, Thunga G, Rashid M (2022). Prevalence and predictors of potentially inappropriate medication Use among Elderly patients using updated Beers Criteria 2019: a single centered retrospective analysis. Curr Drug Saf.

[15] Abdelwahed AA, El-Dahiyat F, Aljawamis D, Al Ajimi J, Bin Rafeea KJ (2021). Potentially inappropriate medications in older adults according to Beers criteria 2019: prevalence and risk factors. Int J Clin Pract.

[16] Walker BS, Collier BR, Bower KL, Lollar DI, Faulks ER, Matos M (2019). The prevalence of Beers Criteria Medication Use and associations with Falls in geriatric patients at a level 1 Trauma Center. Am Surg.

[17] Dills H, Shah K, Messinger-Rapport B, Bradford K, Syed Q (2018). Deprescribing Medications for Chronic Diseases Management in Primary Care settings: a systematic review of Randomized controlled trials. J Am Med Dir Assoc.

[18] Gray SL, Hart LA, Perera S, Semla TP, Schmader KE, Hanlon JT (2018). Meta-analysis of interventions to reduce adverse drug reactions in older adults. J Am Geriatr Soc.

[19] Cooper JA, Cadogan CA, Patterson SM, Kerse N, Bradley MC, Ryan C (2015). Interventions to improve the appropriate use of polypharmacy in older people: a Cochrane systematic review. BMJ Open.

[20] PPA. Pediatric Pharmacy Association [Internet]. [cited 2024 May 13]. https://www.ppag.org/.

[21] Meyers RS, Thackray J, Matson KL, McPherson C, Lubsch L, Hellinga RC, et al. Key potentially I nappropriate D rugs in pediatrics: the KIDs list. J Pediatr Pharmacol Ther. 2020;25(3):175–91. 10.5863/1551-6776-25.3.17510.5863/1551-6776-25.3.175. 10.5863/1551-6776-25.3.175 PMC7134587 32265601

[22] Department of Pediatrics Tawam Hospital. - Sheikh Hamdan Bin Rashid Al Maktoum Award for Medical Sciences - HMA [Internet]. [cited 2024 May 13]. http://hmaward.org.ae/profile.php?id=1520.

[23] Daele MC, Jaeken J, Schueren PVD, Zimmerman A, Bon PVD. Dystonic reactions in children caused by Metoclopramide. Arch Dis Child. 1970;45(239):130. 10.1136/adc.45.239.130. 10.1136/adc.45.239.130 PMC2020385 5440179

[24] Research C. for DE and. FDA Drug Safety Communication: FDA evaluating the risks of using the pain medicine tramadol in children aged 17 and younger. FDA. 2019; https://www.fda.gov/drugs/drug-safety-and-availability/fda-drug-safety-communication-fda-evaluating-risks-using-pain-medicine-tramadol-children-aged-17-and.

[25] Amid COVID-19. the importance of developing an positive adverse drug reaction (ADR) and medical device incident (MDI) reporting culture for Global Health and public safety - PubMed [Internet]. [cited 2023 Mar 10]. https://pubmed.ncbi.nlm.nih.gov/32454982/. 10.1186/s40545-020-00219-1 PMC7233677 32454982

[26] Anderson VH, Anderson J, Durham S, Collard E (2022). Evaluation and implementation of KIDs list recommendations in a University Health System. J Pediatr Pharmacol Ther.

[27] Metoclopramide-containing medicines - referral | European Medicines Agency [Internet]. 2013. https://www.ema.europa.eu/en/medicines/human/referrals/metoclopramide-containing-medicines.

[28] Morris AD, Chen J, Lau E, Poh J, Domperidone-Associated QT (2016). Interval prolongation in non-oncologic Pediatric patients: a review of the literature. Can J Hosp Pharm.

[29] Cheng A (2011). Emergency department use of oral ondansetron for acute gastroenteritis-related vomiting in infants and children. Paediatr Child Health.

[30] Albayrak A, Başgut B, Bıkmaz GA, Karahalil B (2022). Clinical pharmacist assessment of drug-related problems among intensive care unit patients in a Turkish university hospital. BMC Health Serv Res.

[31] Viktil KK, Blix HS (2008). The impact of clinical pharmacists on drug-related problems and clinical outcomes. Basic Clin Pharmacol Toxicol.

[32] Parrish RH, Casher D, van den Anker J, Benavides S (2019). Creating a Pharmacotherapy Collaborative Practice Network To Manage Medications for Children and Youth: a Population Health Perspective. Child (Basel).

